# Bis[2-(pyridin-2-yl)ethanol-κ^2^ *N*,*O*]bis­(thio­cyanato-κ*N*)nickel(II)

**DOI:** 10.1107/S1600536810046647

**Published:** 2010-11-17

**Authors:** Lei Lv, Xiumin Qiu, Jie Yang, Shizheng Liu, Dacheng Li

**Affiliations:** a School of Chemistry and Chemical Engineering, Liaocheng University, Shandong 252059, People’s Republic of China

## Abstract

In the title complex, [Ni(NCS)_2_(C_7_H_9_NO)_2_], the Ni^II^ atom is in a distorted octa­hedral coordination environment defined by two N atoms of the two thio­cyanate ions and by the N and O atoms of the two chelating 2-(pyridin-2-yl)ethanol ligands. The complex mol­ecule is located around a crystallographic inversion center. In the crystal, mol­ecules are connected into a two-dimensional polymeric structure parallel to (100) by O—H⋯S hydrogen bonds.

## Related literature

For related structures, see: Pan *et al.* (2007 ▶); Yu *et al.* (2010 ▶).
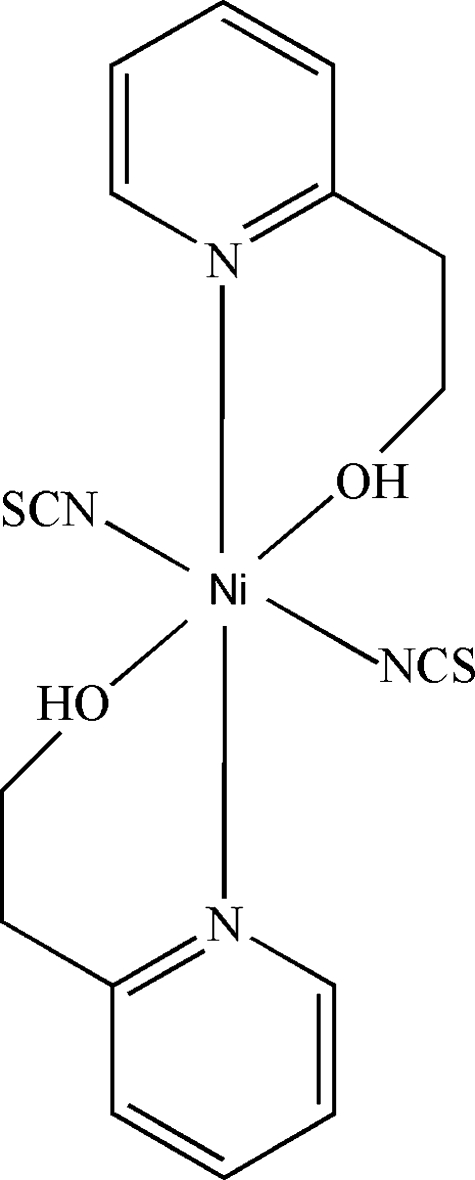


## Experimental

### 

#### Crystal data

[Ni(NCS)_2_(C_7_H_9_NO)_2_]
*M* *_r_* = 421.17
Monoclinic, 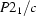

*a* = 8.7197 (9) Å
*b* = 13.8634 (15) Å
*c* = 7.8655 (7) Å
β = 105.496 (2)°
*V* = 916.26 (16) Å^3^
*Z* = 2
Mo *K*α radiation
μ = 1.30 mm^−1^
*T* = 298 K
0.42 × 0.41 × 0.40 mm

#### Data collection

Bruker SMART 1000 CCD diffractometer
Absorption correction: multi-scan (*SADABS*; Sheldrick, 1996 ▶) *T*_min_ = 0.611, *T*_max_ = 0.624
4493 measured reflections
1616 independent reflections
1408 reflections with *I* > 2σ(*I*)
*R*_int_ = 0.020

#### Refinement

*R*[*F* ^2^ > 2σ(*F* ^2^)] = 0.027
*wR*(*F* ^2^) = 0.076
*S* = 1.07
1616 reflections
115 parameters
5 restraints
H-atom parameters constrained
Δρ_max_ = 0.41 e Å^−3^
Δρ_min_ = −0.55 e Å^−3^

### 

Data collection: *SMART* (Siemens, 1996 ▶); cell refinement: *SAINT* (Siemens, 1996 ▶); data reduction: *SAINT*; program(s) used to solve structure: *SHELXS97* (Sheldrick, 2008 ▶); program(s) used to refine structure: *SHELXL97* (Sheldrick, 2008 ▶); molecular graphics: *SHELXTL* (Sheldrick, 2008 ▶); software used to prepare material for publication: *SHELXTL*.

## Supplementary Material

Crystal structure: contains datablocks I, global. DOI: 10.1107/S1600536810046647/gk2318sup1.cif

Structure factors: contains datablocks I. DOI: 10.1107/S1600536810046647/gk2318Isup2.hkl

Additional supplementary materials:  crystallographic information; 3D view; checkCIF report

## Figures and Tables

**Table 1:** Selected bond lengths (Å)

Ni1—N2	2.052 (2)
Ni1—N1	2.1011 (19)
Ni1—O1	2.1030 (15)

**Table 2:** Hydrogen-bond geometry (Å, °)

*D*—H⋯*A*	*D*—H	H⋯*A*	*D*⋯*A*	*D*—H⋯*A*
O1—H1⋯S1^i^	0.93	2.66	3.2183 (19)	119

Symmetry code: (i) 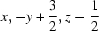.

**Fig. 1.**
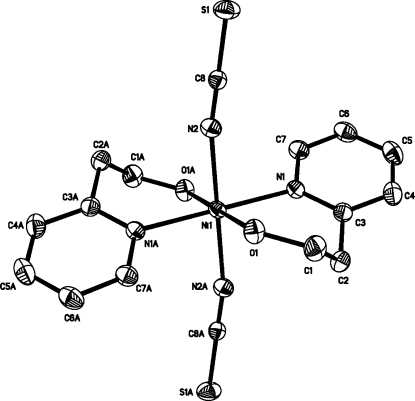
The molecular structure of the title compound, with atom labels and 30% probability displacement ellipsoids. Atoms labelled with the suffix A are generated by the symmetry operation -x + 2,-y,-z.

## supplementary crystallographic information

### Comment

Molecular materials with porous coordination frameworks have recently drawn considerable interest because of their attractive properties. The importance of the work in this area is the construction of porous materials from metal ions and organic ligands as building blocks. As a flexible ligand, 2-(hydroxyethyl)pyridine (hepH) can adopt a variety of possible binding modes. As a contribution to this field, we report here the synthesis and structure of the title compound.

In the title complex molecule, [Ni(SCN)_2_(C_7_H_9_NO)_2_], the nickel(II) atom displays a distorted octahedral coordination geometry, provided by two N atoms of two thiocyanate ions and by the N and O atoms of two 2-(hydroxyethyl)pyridine ligands. Bond lengths and angles involving the metal centre are typical and comparable with those observed in related Co^(II)^ complexes (Pan *et al.*, 2007; Yu *et al.*, 2010). In the crystal structure, molecules are linked through intermolecular O—H···S hydrogen bonds (Table 1).

### Experimental

To a stirred methanol (10 ml) and acetonitrile (10 ml) solution of NiCl_2_.6H_2_O (1 mmol, 238 mg) was added 2-(pyridyn-2-yl)ethanol (2 mmol, 246 mg) in 5 ml me thanol and tetramethylammonium hydroxide(0.4 mmol, 165 mg, 25% solution in water). After 30 min to the above solution was added KSCN (2 mmol, 194 mg) and the solution was stirred for additional 6 h. The resulting red solution was filtered and was allowed to stand at room temperature for about one week, whereupon blue block crystal, suitable for X-ray diffraction analysis, was obtained.

### Refinement

All H atoms were placed in geometrically idealized positions (O–H= 0.93, C—H = 0.93-0.97 Å) and treated as riding on their parent atoms, with *U*_iso_(H) = 1.2*U*_eq_(C). A rigid bond restraints were applied to the Uij values of Ni1, N2, S1 and C8 atoms *via* DELU instruction of SHELXL97 (Sheldrick, 2008).

### Crystal data

**Table:**

[Ni(NCS)_2_(C_7_H_9_NO)_2_]	*F*(000) = 436
*M**_r_* = 421.17	*D*_x_ = 1.527 Mg m^−^^3^
Monoclinic, *P*2_1_/*c*	Mo *K*α radiation, λ = 0.71073 Å
Hall symbol: -P 2ybc	Cell parameters from 2904 reflections
*a* = 8.7197 (9) Å	θ = 2.4–28.0°
*b* = 13.8634 (15) Å	µ = 1.30 mm^−^^1^
*c* = 7.8655 (7) Å	*T* = 298 K
β = 105.496 (2)°	Block, blue
*V* = 916.26 (16) Å^3^	0.42 × 0.41 × 0.40 mm
*Z* = 2	

### Data collection

**Table:**

Bruker SMART 1000 CCD diffractometer	1616 independent reflections
Radiation source: fine-focus sealed tube	1408 reflections with *I* > 2σ(*I*)
graphite	*R*_int_ = 0.020
phi and ω scans	θ_max_ = 25.0°, θ_min_ = 2.4°
Absorption correction: multi-scan (*SADABS*; Sheldrick, 1996)	*h* = −10→8
*T*_min_ = 0.611, *T*_max_ = 0.624	*k* = −16→15
4493 measured reflections	*l* = −7→9

### Refinement

**Table:**

Refinement on *F*^2^	Primary atom site location: structure-invariant direct methods
Least-squares matrix: full	Secondary atom site location: difference Fourier map
*R*[*F*^2^ > 2σ(*F*^2^)] = 0.027	Hydrogen site location: inferred from neighbouring sites
*wR*(*F*^2^) = 0.076	H-atom parameters constrained
*S* = 1.07	*w* = 1/[σ^2^(*F*_o_^2^) + (0.0356*P*)^2^ + 0.5792*P*] where *P* = (*F*_o_^2^ + 2*F*_c_^2^)/3
1616 reflections	(Δ/σ)_max_ = 0.001
115 parameters	Δρ_max_ = 0.41 e Å^−^^3^
5 restraints	Δρ_min_ = −0.55 e Å^−^^3^

### Fractional atomic coordinates and isotropic or equivalent isotropic displacement parameters (Å^2^)

**Table:**

	*x*	*y*	*z*	*U*_iso_*/*U*_eq_	
Ni1	1.0000	0.5000	0.0000	0.03039 (15)	
S1	1.22010 (9)	0.69823 (5)	0.50929 (9)	0.0528 (2)	
N1	0.7906 (2)	0.54485 (14)	0.0607 (2)	0.0341 (4)	
N2	1.1214 (2)	0.57961 (15)	0.2136 (3)	0.0409 (5)	
O1	0.9851 (2)	0.62603 (12)	−0.1523 (2)	0.0415 (4)	
H1	1.0793	0.6489	−0.1717	0.050*	
C1	0.8438 (3)	0.6798 (2)	−0.2289 (4)	0.0498 (7)	
H1A	0.8409	0.6968	−0.3493	0.060*	
H1B	0.8448	0.7391	−0.1630	0.060*	
C2	0.6971 (3)	0.62185 (19)	−0.2278 (3)	0.0452 (6)	
H2A	0.6039	0.6570	−0.2936	0.054*	
H2B	0.7005	0.5613	−0.2886	0.054*	
C3	0.6788 (3)	0.60033 (17)	−0.0472 (3)	0.0373 (5)	
C4	0.5510 (3)	0.6363 (2)	0.0063 (4)	0.0484 (6)	
H4	0.4762	0.6754	−0.0692	0.058*	
C5	0.5344 (3)	0.6142 (2)	0.1710 (4)	0.0535 (7)	
H5	0.4484	0.6374	0.2077	0.064*	
C6	0.6478 (3)	0.5570 (2)	0.2799 (4)	0.0499 (7)	
H6	0.6397	0.5409	0.3920	0.060*	
C7	0.7733 (3)	0.52394 (19)	0.2214 (3)	0.0415 (6)	
H7	0.8496	0.4854	0.2963	0.050*	
C8	1.1618 (3)	0.62905 (16)	0.3352 (3)	0.0320 (5)	

### Atomic displacement parameters (Å^2^)

**Table:**

	*U*^11^	*U*^22^	*U*^33^	*U*^12^	*U*^13^	*U*^23^
Ni1	0.0355 (2)	0.0261 (2)	0.0306 (2)	0.00073 (16)	0.01065 (17)	−0.00126 (16)
S1	0.0696 (5)	0.0394 (4)	0.0428 (4)	−0.0032 (3)	0.0034 (3)	−0.0099 (3)
N1	0.0367 (10)	0.0311 (10)	0.0352 (10)	0.0005 (8)	0.0107 (8)	−0.0025 (8)
N2	0.0445 (11)	0.0363 (11)	0.0409 (10)	−0.0004 (9)	0.0100 (9)	−0.0065 (8)
O1	0.0447 (9)	0.0338 (9)	0.0486 (10)	0.0025 (7)	0.0170 (8)	0.0102 (7)
C1	0.0591 (17)	0.0436 (15)	0.0482 (15)	0.0122 (12)	0.0168 (13)	0.0162 (12)
C2	0.0467 (15)	0.0452 (15)	0.0402 (13)	0.0105 (12)	0.0057 (11)	0.0042 (11)
C3	0.0365 (12)	0.0326 (12)	0.0409 (13)	−0.0014 (10)	0.0070 (10)	−0.0048 (10)
C4	0.0398 (13)	0.0425 (15)	0.0630 (17)	0.0045 (12)	0.0137 (12)	−0.0034 (12)
C5	0.0480 (15)	0.0498 (16)	0.0713 (19)	−0.0007 (13)	0.0310 (14)	−0.0105 (14)
C6	0.0558 (16)	0.0530 (16)	0.0485 (15)	−0.0084 (13)	0.0271 (13)	−0.0068 (13)
C7	0.0453 (14)	0.0429 (14)	0.0393 (13)	−0.0025 (11)	0.0163 (11)	0.0006 (11)
C8	0.0324 (12)	0.0273 (11)	0.0361 (11)	0.0002 (9)	0.0087 (9)	0.0011 (7)

### Geometric parameters (Å, °)

**Table:**

Ni1—N2	2.052 (2)	C2—C3	1.501 (3)
Ni1—N1	2.1011 (19)	C2—H2A	0.9700
Ni1—O1	2.1030 (15)	C2—H2B	0.9700
S1—C8	1.637 (2)	C3—C4	1.386 (4)
N1—C7	1.344 (3)	C4—C5	1.375 (4)
N1—C3	1.349 (3)	C4—H4	0.9300
N2—C8	1.153 (3)	C5—C6	1.374 (4)
O1—C1	1.428 (3)	C5—H5	0.9300
O1—H1	0.9300	C6—C7	1.375 (4)
C1—C2	1.513 (4)	C6—H6	0.9300
C1—H1A	0.9700	C7—H7	0.9300
C1—H1B	0.9700		
			
N2^i^—Ni1—N2	180.00 (9)	O1—C1—H1B	109.5
N2^i^—Ni1—N1^i^	86.82 (8)	C2—C1—H1B	109.5
N2—Ni1—N1^i^	93.18 (8)	H1A—C1—H1B	108.1
N2^i^—Ni1—N1	93.18 (8)	C3—C2—C1	114.5 (2)
N2—Ni1—N1	86.82 (8)	C3—C2—H2A	108.6
N1^i^—Ni1—N1	180.0	C1—C2—H2A	108.6
N2^i^—Ni1—O1	92.32 (7)	C3—C2—H2B	108.6
N2—Ni1—O1	87.68 (7)	C1—C2—H2B	108.6
N1^i^—Ni1—O1	92.38 (7)	H2A—C2—H2B	107.6
N1—Ni1—O1	87.62 (7)	N1—C3—C4	121.2 (2)
N2^i^—Ni1—O1^i^	87.68 (7)	N1—C3—C2	117.8 (2)
N2—Ni1—O1^i^	92.32 (7)	C4—C3—C2	120.9 (2)
N1^i^—Ni1—O1^i^	87.62 (7)	C5—C4—C3	120.1 (3)
N1—Ni1—O1^i^	92.38 (7)	C5—C4—H4	119.9
O1—Ni1—O1^i^	180.00 (5)	C3—C4—H4	119.9
C7—N1—C3	118.1 (2)	C6—C5—C4	118.5 (3)
C7—N1—Ni1	118.18 (16)	C6—C5—H5	120.8
C3—N1—Ni1	123.47 (16)	C4—C5—H5	120.8
C8—N2—Ni1	167.28 (19)	C5—C6—C7	119.2 (3)
C1—O1—Ni1	126.06 (15)	C5—C6—H6	120.4
C1—O1—H1	117.0	C7—C6—H6	120.4
Ni1—O1—H1	117.0	N1—C7—C6	122.8 (3)
O1—C1—C2	110.9 (2)	N1—C7—H7	118.6
O1—C1—H1A	109.5	C6—C7—H7	118.6
C2—C1—H1A	109.5	N2—C8—S1	179.3 (2)
			
N2^i^—Ni1—N1—C7	121.90 (18)	N1—Ni1—O1—C1	−23.9 (2)
N2—Ni1—N1—C7	−58.10 (18)	O1—C1—C2—C3	65.5 (3)
O1—Ni1—N1—C7	−145.91 (18)	C7—N1—C3—C4	1.0 (3)
O1^i^—Ni1—N1—C7	34.09 (18)	Ni1—N1—C3—C4	−173.23 (18)
N2^i^—Ni1—N1—C3	−63.84 (19)	C7—N1—C3—C2	−178.9 (2)
N2—Ni1—N1—C3	116.16 (19)	Ni1—N1—C3—C2	6.9 (3)
O1—Ni1—N1—C3	28.35 (18)	C1—C2—C3—N1	−63.6 (3)
O1^i^—Ni1—N1—C3	−151.65 (18)	C1—C2—C3—C4	116.5 (3)
N1^i^—Ni1—N2—C8	176.2 (9)	N1—C3—C4—C5	−1.2 (4)
N1—Ni1—N2—C8	−3.8 (9)	C2—C3—C4—C5	178.7 (2)
O1—Ni1—N2—C8	84.0 (9)	C3—C4—C5—C6	0.7 (4)
O1^i^—Ni1—N2—C8	−96.0 (9)	C4—C5—C6—C7	0.0 (4)
N2^i^—Ni1—O1—C1	69.2 (2)	C3—N1—C7—C6	−0.4 (4)
N2—Ni1—O1—C1	−110.8 (2)	Ni1—N1—C7—C6	174.2 (2)
N1^i^—Ni1—O1—C1	156.1 (2)	C5—C6—C7—N1	−0.1 (4)

Symmetry codes: (i) −*x*+2, −*y*+1, −*z*.

### Hydrogen-bond geometry (Å, °)

**Table:**

*D*—H···*A*	*D*—H	H···*A*	*D*···*A*	*D*—H···*A*
O1—H1···S1^ii^	0.93	2.66	3.2183 (19)	119

Symmetry codes: (ii) *x*, −*y*+3/2, *z*−1/2.
